# Estimation of the post‐mortem interval by modelling the changes in oral bacterial diversity during decomposition

**DOI:** 10.1111/jam.15771

**Published:** 2022-09-09

**Authors:** Xingchun Zhao, Zengtao Zhong, Zichun Hua

**Affiliations:** ^1^ School of Biopharmacy China Pharmaceutical University Nanjing P.R. China; ^2^ National Engineering Laboratory for Forensic Science Beijing P.R. China; ^3^ Institute of Forensic Science Ministry of Public Security Beijing P.R. China; ^4^ Key Laboratory of Forensic Genetics of Ministry of Public Security Beijing P.R. China; ^5^ Department of Microbiology College of Life Sciences Nanjing Agricultural University Nanjing P.R. China

**Keywords:** death time, high‐throughput sequencing, oral cavity flora, post‐mortem interval, SD rats

## Abstract

**Aims:**

Decomposition, a complicated process, depends on several factors, including carrion insects, bacteria and the environment. However, the composition of and variation in oral bacteria over long periods of decomposition remain unclear. The current study aims to illustrate the composition of oral bacteria and construct an informative model for estimating the post‐mortem interval (PMI) during decomposition.

**Methods and Results:**

Samples were collected from rats' oral cavities for 59 days, and 12 time points in the PMI were selected to detect bacterial community structure by sequencing the V3–V4 region of the bacterial 16S ribosomal RNA (16S rRNA) gene on the Ion S5 XL platform. The results indicated that microorganisms in the oral cavity underwent great changes during decomposition, with a tendency for variation to first decrease and then increase at day 24. Additionally, to predict the PMI, an informative model was established using the random forest algorithm. Three genera of bacteria (*Atopostipes*, *Facklamia* and *Cerasibacillus*) were linearly correlated at all 12 time points in the 59‐day period. Planococcaceae was selected as the best feature for the last 6 time points. The *R* ^2^ of the model reached 93.94%, which suggested high predictive accuracy. Furthermore, to predict the functions of the oral microbiota, PICRUSt results showed that energy metabolism was increased on day 3 post‐mortem and carbohydrate metabolism surged significantly on days 3 and 24 post‐mortem.

**Conclusions:**

Overall, our results suggested that post‐mortem oral microbial community data can serve as a forensic resource to estimate the PMI over long time periods.

**Significance and Impact of the Study:**

The results of the present study are beneficial for estimating the PMI. Identifying changes in the bacterial community is of great significance for further understanding the applicability of oral flora in forensic medicine.

## INTRODUCTION

The microbiome plays multiple roles in human health in clinic settings and is widely used in human health evaluations (Consortium, 2012). There are more than 700 bacterial species in the oral cavity (Gholizadeh et al., 2016). The representative taxa belong to the phyla *Actinobacteria*, *Bacteroidetes*, *Firmicutes*, *Proteobacteria*, *Spirochaetes*, *Synergistes* and *Tenericutes* (Dewhirst et al., 2010). Researchers are committed to studying the synergetic relationship between bacteria and host disease (Duran‐Pinedo et al., 2014). However, the understanding of the changes in oral bacteria after death is insufficient.

The decomposition process is variable and complex and is affected by many factors, among which the microbial community is particularly important (Byrd & Castner, 2019). The decomposition of a corpse is usually divided into four stages: fresh, initial decomposition, progressive decomposition and skeletonization (Payne, 1965). Previous studies predicted a dynamic and arbitrary microbial community that is affected by diffusion, local expansion and competitive interactions after death (Metcalf et al., 2016). A lack of oxygen in the corpse leads to cell autolysis and the release of hydrogen sulfide and methane macromolecules such as cadaveric alkali and putrescine (Debruyn & Hauther, 2017), which results in cadaver inflation and rupture (Metcalf et al., 2013). The above process is mainly caused by autochthonous bacteria (Janaway et al., 2009) and ultimately leads to a transformation and shift from aerobic bacteria to anaerobic bacteria (Melvin et al., 1984). Therefore, microbes play an important role during decomposition (Deel et al., 2021; Mondor & Tremblay, 2012). Oral autochthonous bacteria play a crucial role in the early stages of human decomposition (Janaway et al., 2009; Hu et al., 2021). A large number of studies have been carried out to examine the composition and characteristic changes of the microbial community in the mouth (Hyde et al., 2015; Pechal et al., 2018). In a recent study, Dong et al. (2019) suggested that succession of the oral microbial community could be used to estimate the post‐mortem interval (PMI) in forensic inspection shortly after death. Estimating the PMI is necessary in every death investigation since it can be employed to identify victims or defendants (Liu et al., 2021; Metcalf et al., 2013). At present, some studies focus on the microbial community after death (Speruda et al., 2021), but the estimation of the PMI is still insufficient. Little is known about the succession of oral microbial communities during cadaver decomposition, especially over long time periods.

Here, we developed a model for estimating the cadaveric PMI over long time periods by using oral flora. To generate the model, we explored the succession of oral microorganisms in Sprague–Dawley (SD) rat cadavers up to 59 days of decay in a constant environment. The oral microbial community of the SD rat cadavers was characterized by high‐throughput sequencing analysis of the 16S rRNA gene. In addition, the successional pattern of the oral microbial community associated with corruption was evaluated, and microbial characteristics with potential use as biomarkers for PMI estimation were screened. A random forest regression (RFR) model was constructed to estimate the PMI. Our research contributes to a comprehensive understanding of corpse decomposition, providing an important supplement to previous studies.

## MATERIALS AND METHODS

### Experimental design and sample collection

The procedures involving animal care in this study were approved by the Animal Care and Use Committee of Nanjing Agricultural University (Nanjing, China) (permit number: SYXK(Su)2017‐0007). In this study, 96 male SD rats were obtained from Shanghai SLAC Laboratory Animal Co., Ltd. All rats were housed in separate sterile anti‐scavenging cages (0.29 × 0.2 × 0.19 m) in an animal room with a 12‐h light/dark cycle. After a week of adaptation to the animal room, all rats were humanely euthanized by cervical dislocation. All corpses were placed in a room to mimic the process of human decay. The air temperature ranged from 23 to 26°C (25.18 ± 0.75°C, mean ± SD) and the humidity ranged from 28% to 82% (55.55 ± 14.74%, mean ± SD) during the decay period. The weight loss per rat during decomposition was recorded.

To observe bacterial community composition, oral samples were alternately taken by swabs from day 0 to day 59. The time points were set as 0 h, day 1, day 3, day 5, day 10, day 15, day 20, day 24, day 30, day 40, day 52 and day 59. The groups including eight rats each were defined as AMH0, AMD1, AMD3, AMD5, AMD10, AMD15, AMD20, AMD24, AMD30, AMD40, AMD52 and AMD59, respectively. The sampled areas of the oral cavity included the palate, tongue, inner cheek mucosa, and tooth surface. The swab tips were cut off, preserved in 1.5 ml micro‐tubes and frozen at −20°C until further utilization. A total of 96 samples were collected.

### Bacterial genomic DNA extraction

Following the manufacturer's instructions, total genomic DNA was extracted from the collected swab samples by utilizing a QIAamp DNA Mini Kit (Qiagen) and stored at −80°C. The concentration and purity of the extracted DNA were examined by a NanoDrop 2000 instrument (Thermo Scientific), followed by 1% agarose gel electrophoresis.

### 16S rDNA sequencing

16S rDNA amplicon sequencing was accomplished using the Ion S5 XL sequencing platform. The analyses were completed at Novogene Biological Information Technology Co. According to the manufacturer's protocol, the V3‐V4 region of the 16S rRNA gene was PCR‐amplified with the primers 341F (5′‐CCTAYGGGRBGCASCAG‐3′) and 806R (5′‐GGACTACNNGGTATCTAAT‐3′). Amplicons ranging from 400 to 450 bp in length were selected, and pooled amplicons were single‐end sequenced using SE600 technology. Sequencing libraries were created using the Ion Plus Fragment Library Kit with 48 reactions (Thermo Fisher), followed by quantitation with a Qubit 2.0 fluorometer (Thermo Fisher Scientific) and Agilent Bioanalyzer 2100 system.

### Data analysis

To remove low‐quality parts of reads, barcodes and primers, Cutadapt (V1.9.1) (Langille et al., 2013) was employed. Chimaeras in raw reads were distinguished and removed by the UCHIME algorithm (Edgar et al., 2011). The remaining sequences (clean reads) with ≥97% identity were assigned to operational taxonomic units (OTUs) according to UPARSE (v7.0.1001) (Haas et al., 2011), and the reads with the highest frequency of occurrence were selected as representative sequences. Mothur was adopted to assign taxonomic information (Schloss et al., 2009). The representative OTU sequences were annotated by comparison with the Silva (v132) database. For species annotation, the threshold was set to 0.8–1 to obtain taxonomic information, and the community composition of each sample was determined at each classification level: Kingdom, phylum, class, order, family, genus and species. Sequences were clustered into OTUs with 97% similarity.

Alpha diversity (Chao1, abundance‐based coverage estimator [ACE], Shannon and Simpson indexes) analysis was performed by using QIIME (version 1.9.1; Caporaso et al., 2010). The differences in alpha diversity between groups were compared by the nonparametric Kruskal–Wallis test and Dunn's multiple comparison test. When the *p* < 0.05 after Bonferroni correction, the difference was considered statistically significant.

The beta diversity index was analysed by R software, followed by a nonparametric test. A non‐metric multi‐dimensional scaling (NMDS) diagram was drawn by using R software. The linear discriminant analysis (LDA) effect size (LEfSe) technique was applied to identify the microbial taxa at different time points, which were selected according to an LDA score >2.0 and *p* < 0.05.

### Random forest and regression algorithms for estimating the PMI

Non‐linear regression was conducted in the R environment (v3.6.5). Bacteria in the 96 samples with a relative abundance less than 0.05% were removed. In this study, a random forest model was established with regression trees, and the size of the candidate feature subset was fixed. Random forest was employed to sequence the 30 most abundant OTUs, with six rats in each group as the training set and two rats as the validation set, and then cross‐validation was used to screen the important features. Nine bacteria were screened from 400 bacteria through cross‐validation and then sorted by Random forest and regression algorithms (RFR). To evaluate the performance of the model, the goodness‐of‐fit (*R* ^2^) was calculated (Liu et al., 2020).

### Phylogenetic Investigation of Communities by Reconstruction of Unobserved States

Phylogenetic Investigation of Communities by Reconstruction of Unobserved States (PICRUSt), a software for predicting functional abundances solely based on marker gene sequences, was used to analyse normalized OTU tables. PICRUSt predictions were based on the MetaCyc comprehensive reference database and Enzyme Commission (EC) number database to generate organism‐specific pathways. Kyoto Encyclopedia of Genes and Genomes (KEGG) pathway analysis was conducted with the Pathways module. Based on Fisher's exact test, results with a *p* < 0.01 were defined as significant.

### Statistical analysis

One‐way analysis of variance (ANOVA) with Tukey's multiple comparison test was used to detect noteworthy variance in alpha diversity and beta diversity among sample groups. All statistical analyses were performed in GraphPad Prism (v5.01). A *p* < 0.05 was considered to suggest significance. The bar charts and line charts were drawn in Excel and GraphPad Prism.

## RESULTS

### Visible decay progression of the rats

The air temperature during decomposition ranged from 23 to 26°C (25.18 ± 0.75°C, mean ± SD). The relative humidity ranged from 28% to 82% (55.55 ± 14.74%, mean ± SD). Corpse stiffness began 8 h after death, and the corpse emitted odour. The stiffness disappeared after 16 h, and the odour became stronger. The swelling period occurred on days 3 and 4, while trunk and abdomen gas accumulated, followed by rupture, and decaying liquid flowed out on days 4 and 5. During the period of body decomposition, the daily weight decreased, and a broken line diagram was drawn to show the trend of daily weight loss of each rat (Figure S1).

### The relative abundance of oral cavity microbiota in the rat corpses

By performing high‐throughput sequencing, 7,957,414 raw reads and 7,496,808 clean reads were obtained. The ratio of clean reads to raw reads was 94.21%. The average length of high‐quality sequences was 421 ± 6 bp (mean ± SD). The average number of clean reads per sample was 78091. A total of 947 OTUs were clustered based on 97% similarity, including 925 OTUs annotated to the Silva 132 database (97.67%). The proportions of annotations to phyla, classes, orders, families, genera and species were 97.68%, 95.78, 90.50%, 85.53%, 60.72% and 15.42%, respectively. All of the usable sequences were classified into 18 phyla, 35 classes, 77 orders, 142 families, 273 genera and 120 species. The rarefaction curves and species accumulation boxplot became smooth and stable over time, indicating that sequencing depth and sample quantity met the experimental requirements and reflected the richness of the whole bacterial community (Figure S2).

**FIGURE 1:**
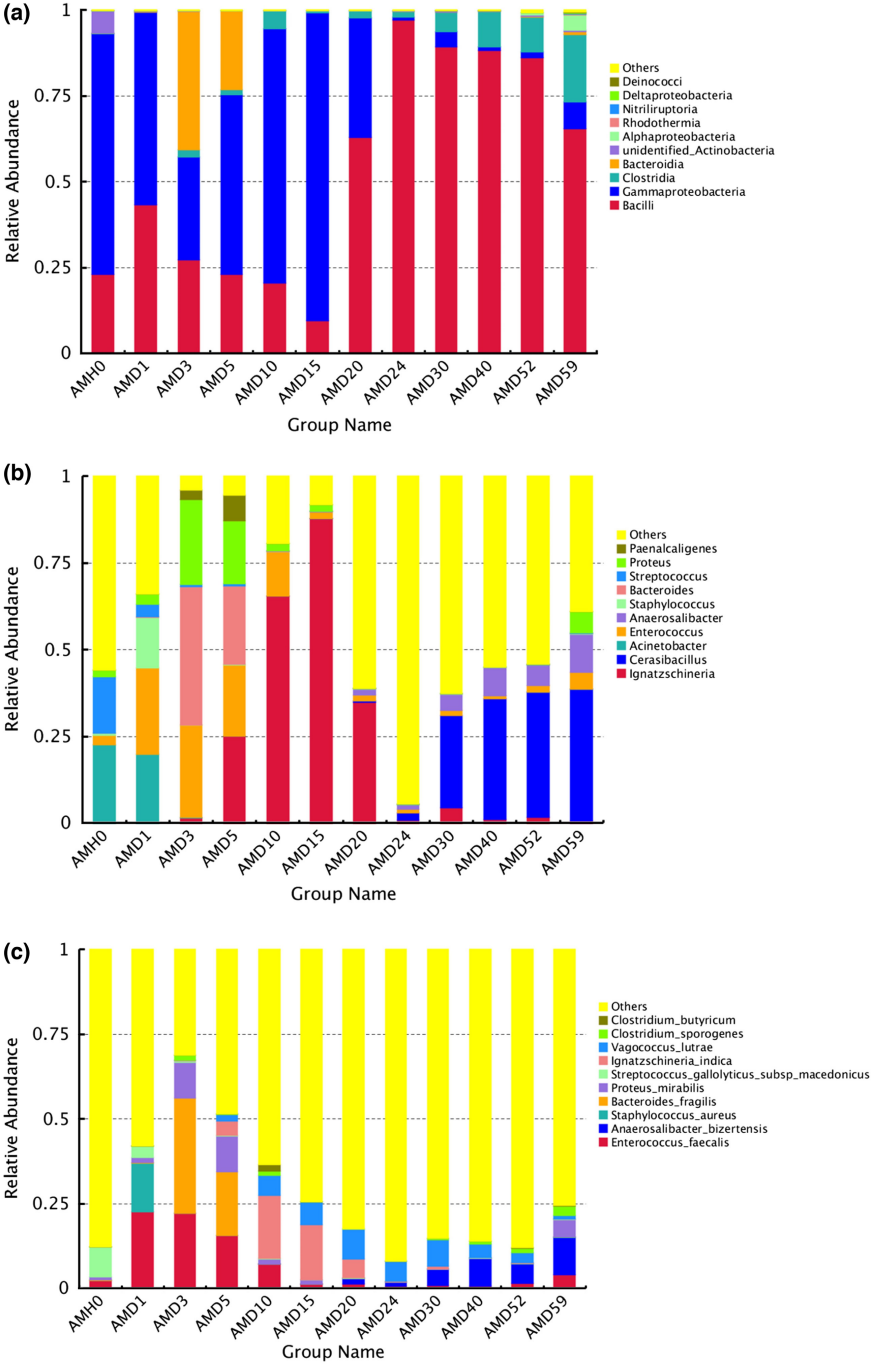
10 most abundant members of the microbial community and their relative abundances at different post‐mortem interval time points at three taxonomic levels: (a) Phylum, (b) family, and (c) genus.

Among the 10 most abundant phyla, *Firmicutes*, *Proteobacteria*, *Bacteroidetes* and *Actinobacteria* were the main phyla in all groups. *Proteobacteria* presented higher relative abundance in the pre‐rupture stage (AMH0, AMD1, AMD3, and AMD5), and *Firmicutes* showed a higher percentage after 20 days, that is, in the late decomposition stage. *Bacteroidetes* and *Actinobacteria* showed a high abundance at AMD3 (40.36%) and AMH0 (6.51%), in the early decay stage, respectively (Figure 1a).

At the genus level, *Ignatzschineria*, *Enterococcus*, *Staphylococcus*, *Proteus* and *Streptococcus* showed different relative abundances among groups. The genus *Acinetobacter* was dominant at AMH0, but it was not present from AMD20 to AMD40. *Enterococcus*, *Bacteroides* and *Proteus* were regarded as the primary bacteria at AMD3 and AMD5 and decreased significantly after 10 days. *Ignatzschineria* and *Cerasibacillus* were much more abundant in the late stage than in the early stage (Figure 1b).

Among the 10 most abundant species, *Enterococcus faecalis* showed a downwards trend, with a larger proportion in the early stage than in the late stage. Notably, *E. faecalis* showed the highest relative abundance at AMD1 (22.62%). The relative abundances of *Bacteroides fragilis* and *Proteus mirabilis* were the highest at AMD3 and AMD5. *Ignatzschineria indica*, *Vagococcus lutrae* and *Anaerosalibacter bizertensis* rapidly proliferated after 10 days and remained stable during the post‐rupture stage (Figure 1c).

### Description of microbial diversity and community configuration

**FIGURE 2:**
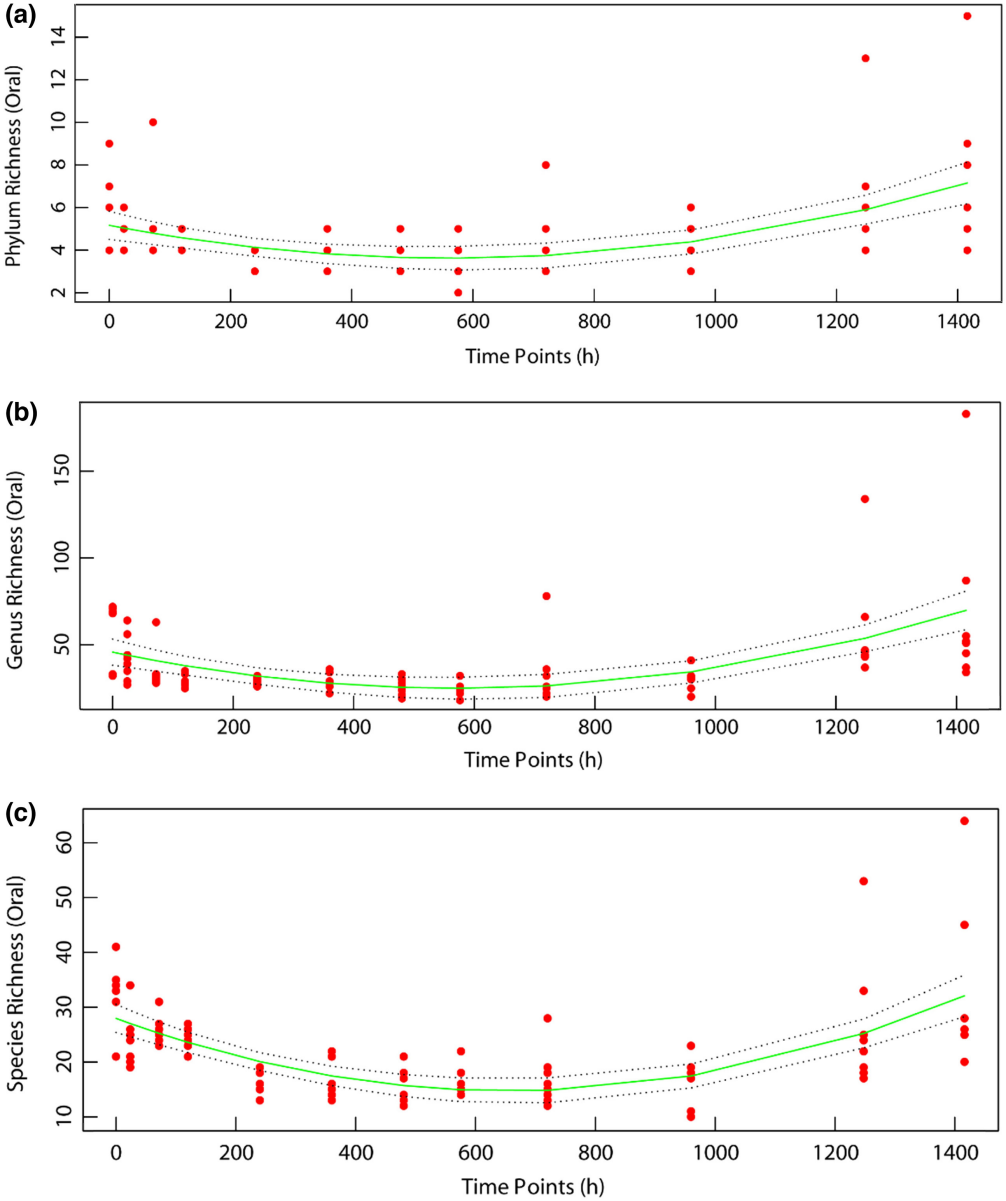
Non‐linear associations of the PMI with bacterial richness at the phylum, genus and species levels.

**FIGURE 3:**
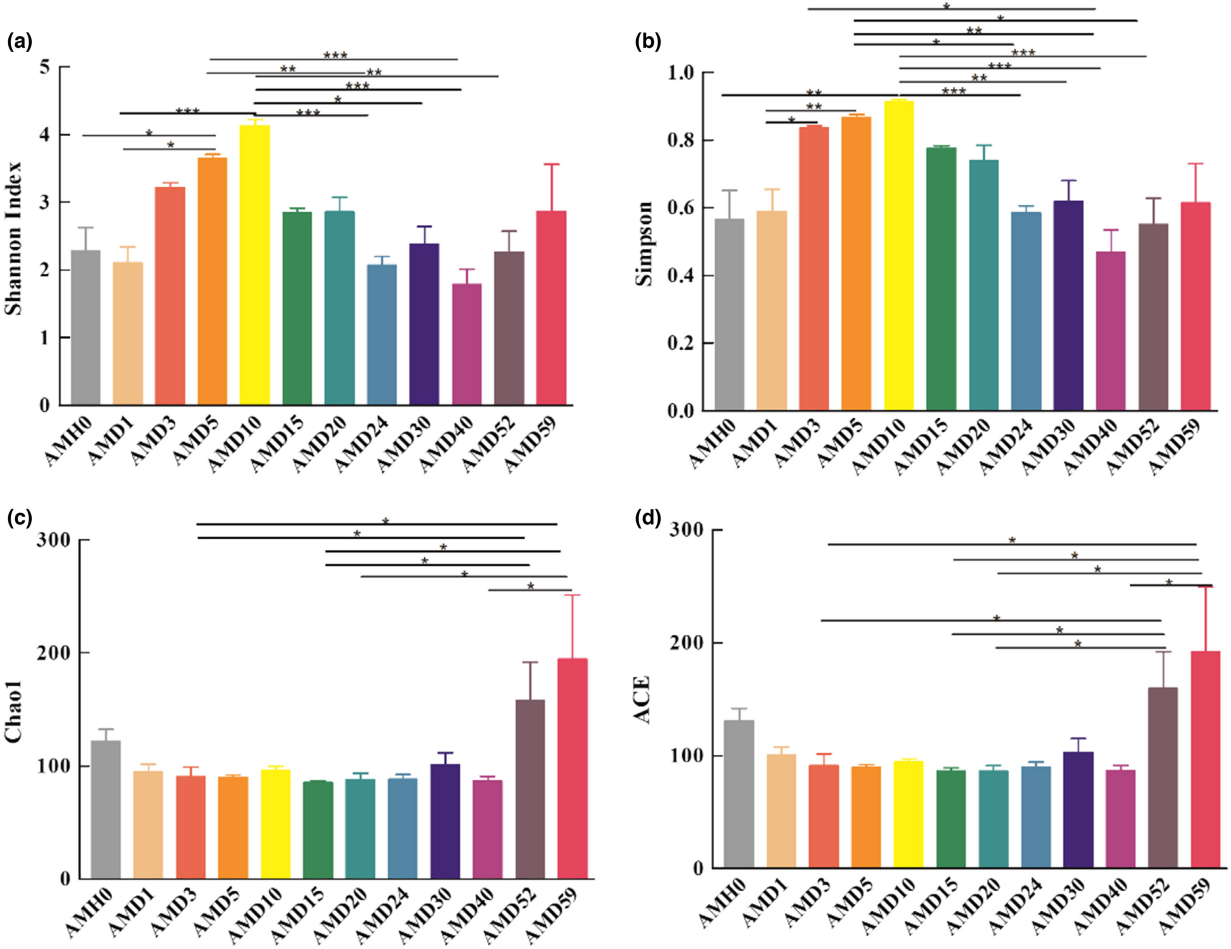
Alpha diversity of the microbial community at 12 time points. Shannon index (a), Simpson index (b), Chao1 index (c), and ACE (d) of 96 samples at 12 time points. *p < 0.001, **p < 0.001, and ***p < 0.001.

To evaluate the richness, evenness and diversity of the oral microbiota community, the Chao1, ACE, Shannon and Simpson indexes were calculated at the level of 97% similarity. The Chao1 and ACE indexes showed a fluctuating trend of bacterial richness before and after 24 days. Univariate nonlinear regression was used to analyse the richness of the bacterial community at the phylum, genus and species levels (Figure 2). The richness index increased before 21 days and decreased after 21 days, with the turning point observed at approximately 21.8 days. During the corpse decomposition process, the peak value of bacterial community diversity occurred at 10 days, and the lowest value was observed at 40 days. After the death of rats, the composition and structure (uniformity) of the oral microbial community both increased. Diversity analysis showed that, compared with that at several PMI time points (AMD24, AMD30, AMD40 and AMD52) in the later stage, the diversity at AMD10 was higher (*p* < 0.05), while the richness showed an upwards trend over time. The comparison among all groups revealed a significant difference in the alpha diversity index (Figure 3).

**FIGURE 4:**
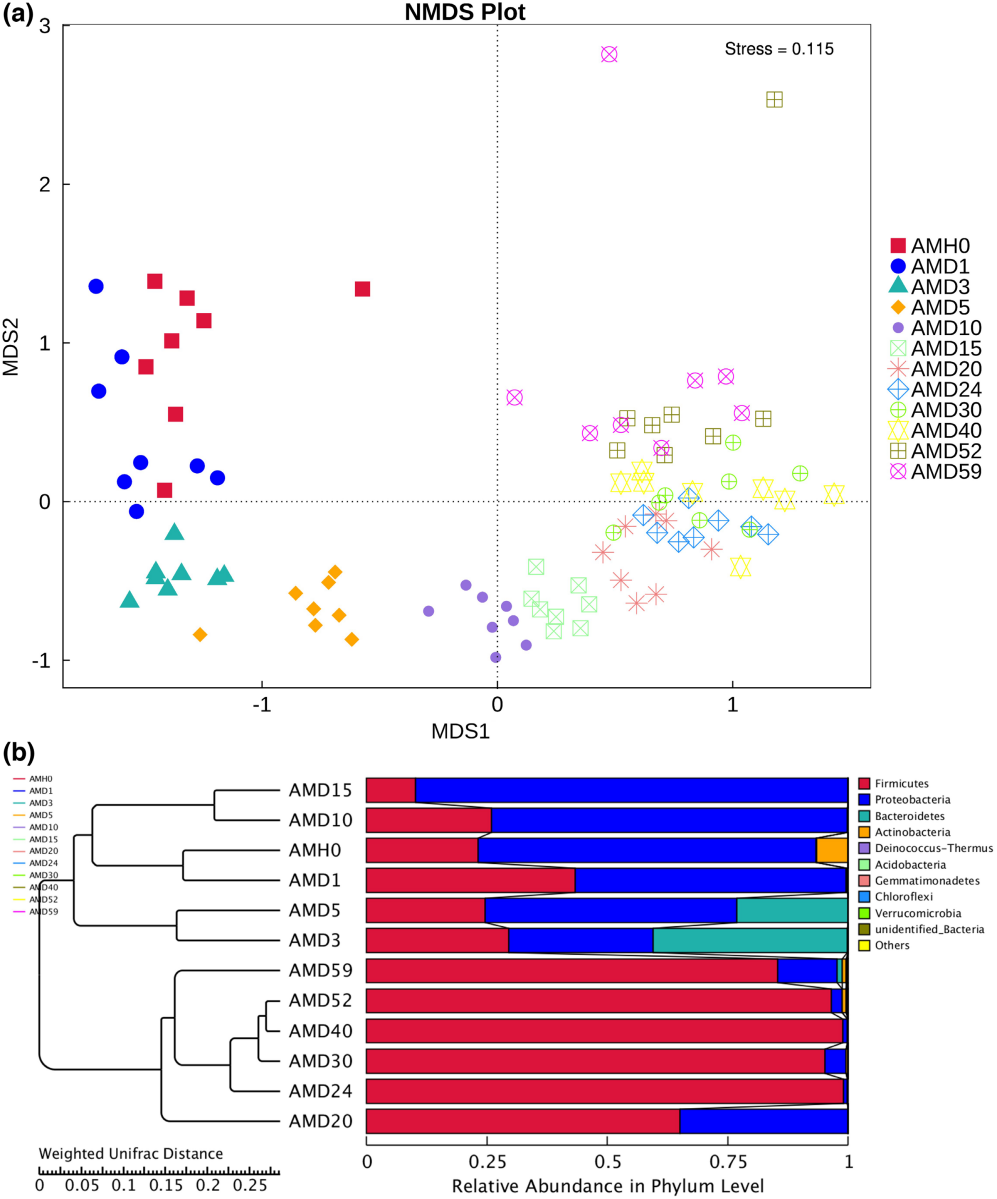
Beta diversity of bacterial communities at 12 time points. (a) NMDS coordination plot of all study subjects. (b) Hierarchical clustering of oral cavity samples based on the weighted UniFrac distance matrix (UPGMA) with bacterial community relative abundances at the phylum level.

According to the NMDS results obtained with Bray–Curtis distances (Figure 4a), the bacterial communities of the oral cavity samples formed 2 discrete clusters corresponding to the early and late stages. The unweighted pair‐group method with arithmetic mean (UPGMA) approach (Figure 4b) was selected to evaluate the weighted UniFrac distance of bacterial relative abundance at the phylum level among groups, and the results were analogous to those of NMDS. In addition, the results showed significant differences between the early and late stages of the PMI, consistent with the results of the above two methods (Table S1).

### The unique microbial communities in the total PMI of this study

**FIGURE 5:**
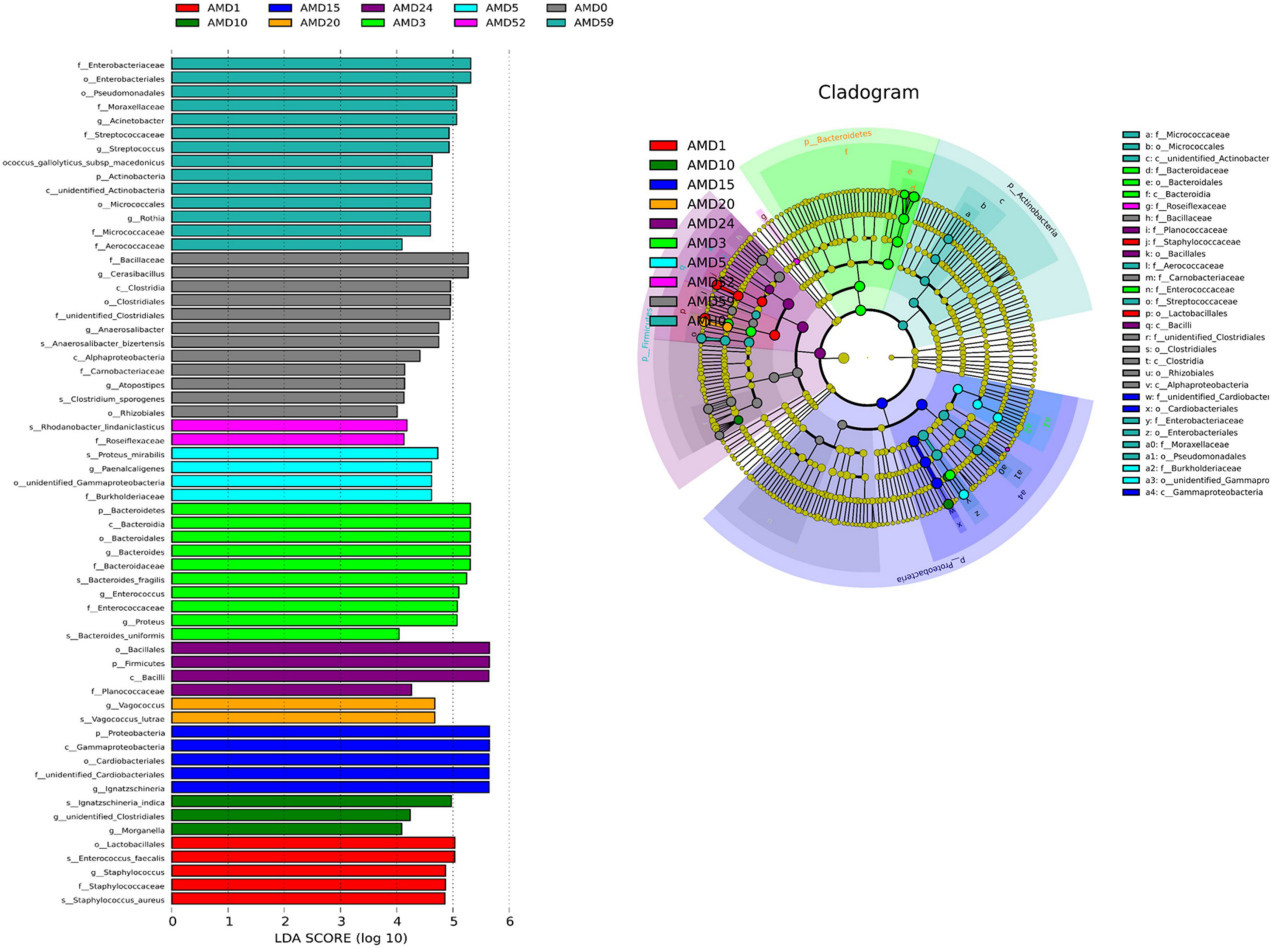
LDA plots of bacteria constructed by the LEfSe technique at different taxonomic levels.

The LEfSe technique was utilized to detect heterogeneous species among groups. As shown in Figure 5a, 12 groups were compared, and 61 species were found in 10 groups. A cladogram displaying the relative abundances of bacterial community members at the phylum, class, order, family, genus, and species levels from inside to outside was constructed (Figure 5b). Enterobacteriaceae, *Lactobacilles, Bacteroidetes*, *P. mirabilis*, *I. indica*, *Gammaproteobacteria*, *Vagococcus lutrea*, *Bacillales*, *Rhodanobacter lindaniclasticus*, and *Cerabacillus* were regarded as crucial bacteria at AMH0, AMD1, AMD3, AMD5, AMD10, AMD15, AMD20, AMD24, AMD52, and AMD59. The bacterial phyla, genera and species were ranked based on perceived importance. *Proteobacteria*, *Firmicutes*, *Bacteroidetes*, and *Actinobacteria* were the four most significant phyla for estimating the PMI. The results for genera showed that two obligate anaerobes (*Cerasibacillus* and *Anaerosalibacter*) were the most critical, followed by *Proteus* and *Ignatzschineria*. At the species level, *A. bizertensis* was preferred, followed by *P. mirabilis*, *V. lutrae*, *B. fragilis*, *I. indica* and *E. faecalis* (Figure S3).

### Constructing a model of bacteria in the oral cavity for estimating the PMI

**FIGURE 6:**
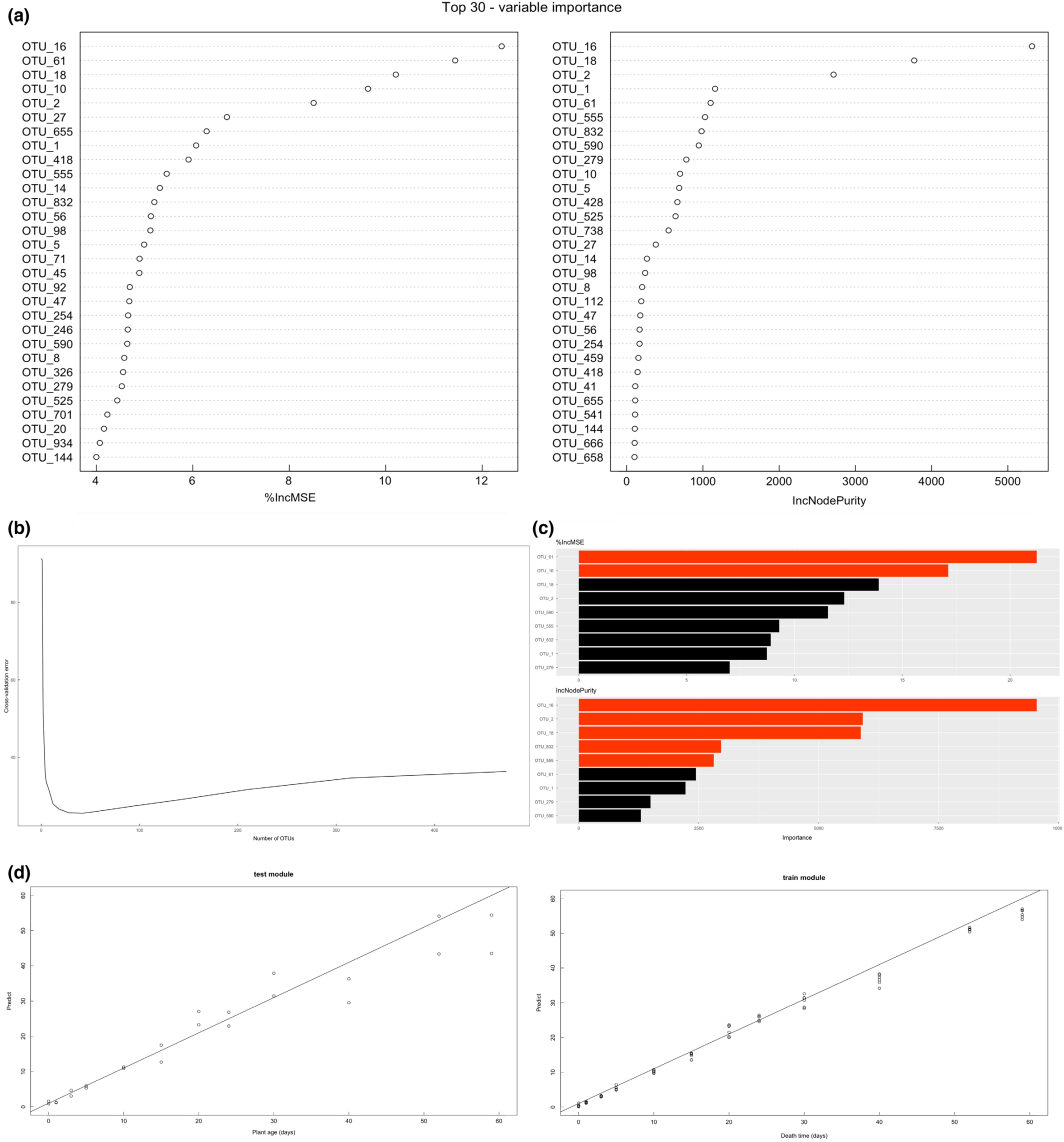
The 30 most abundant OTUs ranked by random forest in each group by using 6 rats as the training set and 2 rats as the validation set. (a) Bacteria with a relative abundance less than 0.05% were removed from 400 samples, and the OTUs ranking in the top 30 were sorted by random forest. (b) Nine bacteria were selected from 400 bacteria by cross‐validation, and the importance of nine bacteria was ranked by random forest. (c) Red represents OTUs with significant differences, and black represents OTUs without significant differences. (d) 6 rats were used as the training set, and 2 rats were used as the validation set.

To explore the potential relationship between bacteria and the PMI at the phylum, genus and species levels, RFR was applied (Figure S4). The 30 most abundant OTUs were sorted by RFR, with six rats in each group as the training set and two rats as the validation set. Nine bacteria were screened from 400 bacteria through cross‐validation and then sorted by RFR (Figure 6).

**FIGURE 7:**
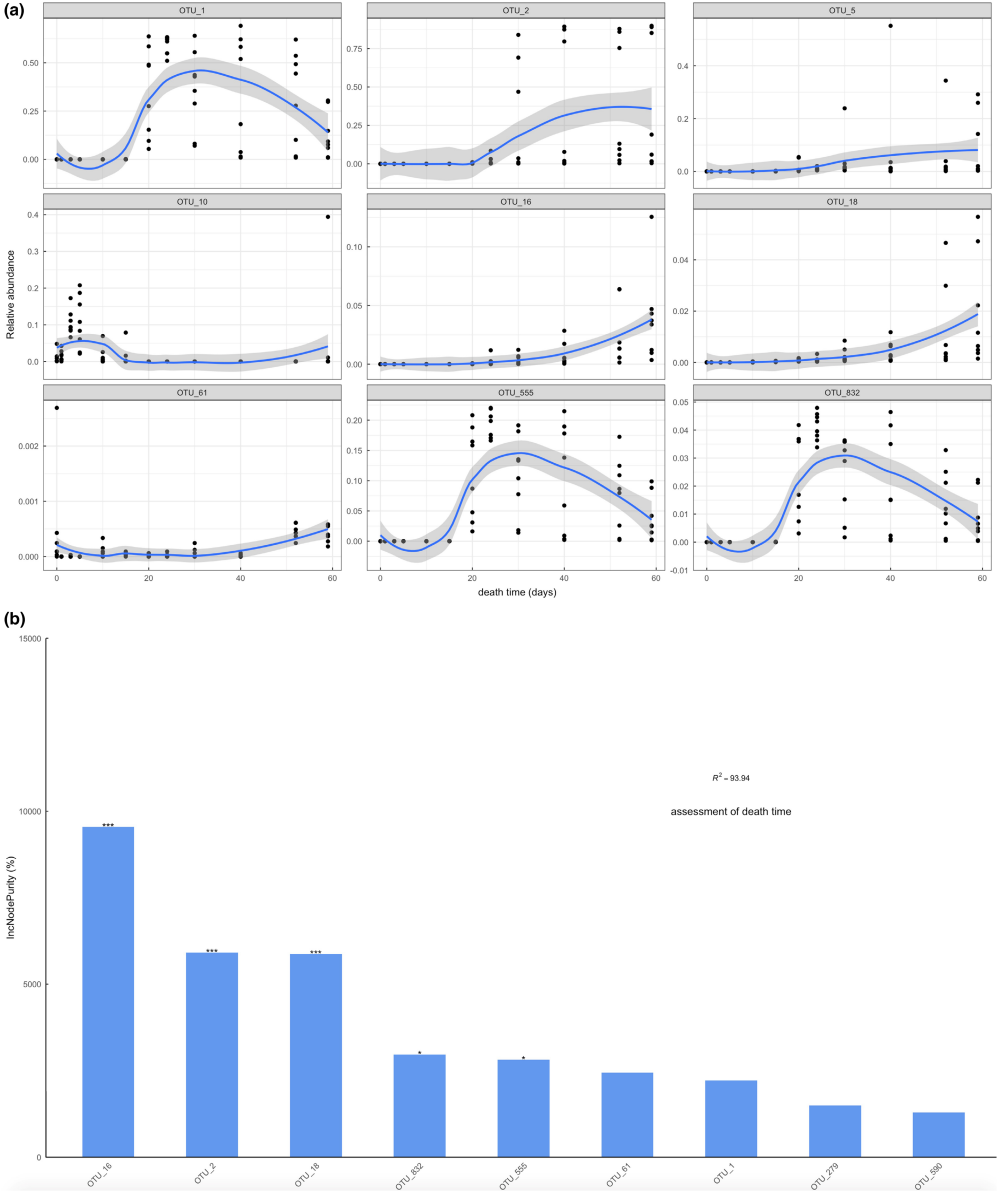
The regression model established by nine kinds of bacteria. (a) The curve of the regression model. OTU16, OTU18, OTU61 and OTU59 were linearly correlated at 12 time points. The other 5 OTUs (OTU1, OTU2, OTU279, OTU555, and OTU832) showed a significant linear relationship with each time point before 20 days. (b) The significance of the IncNodePurity of 9 OTUs was analysed. The R2 value reached 93.94%.

The results showed that OTU16, OTU18, OTU61 and OTU590 were linearly correlated at all 12 time points in the period of 59 days. OTU16 corresponded to Carnobacteriaceae at the family level, and OTU18 and OTU590 corresponded to *Atopostipes* at the genus level. The results indicated that Carnobacteriaceae and *Atopostipes* played an important role in the corruption process. In addition, OTU61 corresponded to *Facklamia* at the genus level. Furthermore, there were five OTUs (OTU1, OTU2, OTU279, OTU555, and OTU832) with a linear relationship with the last 6 time points. OTU2 corresponded to Carnobacteriaceae at the family level and *Cerasibacillus* at the genus level. OTU279 corresponded to Planococcaceae at the family level. These OTUs were selected as the best features to develop the model (Figure 7). The *R* ^2^ of the model reached 93.94%, which suggested high predictive accuracy.

### The functions of the oral microbiota predicted by using PICRUSt

**FIGURE 8:**
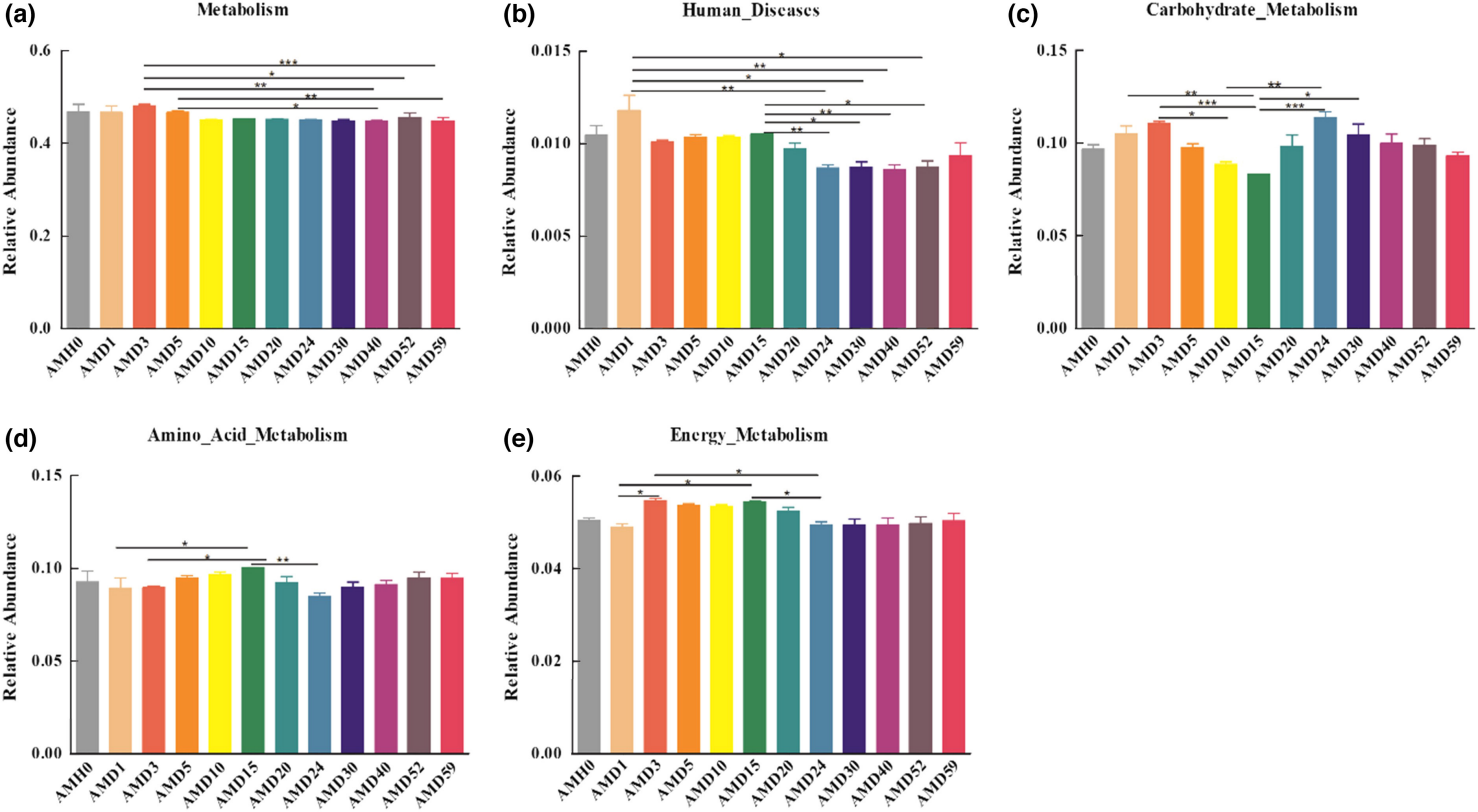
Predicted function of oral cavity flora at 12 PMI time points. The relative abundance of five categories of KEGG pathways at levels 1 (a) and 2 (b, c, d, e) in different groups, *p < 0.05, **p < 0.01, ***p < 0.001.

Changes in the oral cavity flora are related to the modifications of certain nutrient and metabolic pathways. PICRUSt was used to explore the relationships between buccal cavity flora and metabolic pathways. A total of 4516 predicted KOs (KEGG orthology terms) were obtained, and the main signalling pathways were related to metabolism at KEGG level 1, including metabolism, genetic information processing, environmental information processing, Cellular processes, human diseases and organismal systems. At KEGG level 2, carbohydrate metabolism and amino acid metabolism showed the opposite tendency throughout the decomposition process. The variation in energy metabolism at the early stage was consistent with that in carbohydrate metabolism. Genes related to metabolism and human diseases exhibited a higher abundance at the early stage than at the late stage (Figure 8).

## DISCUSSION

As an important method in forensic medicine, estimating the time of death has been the subject of a large number of studies, but its accuracy and applicability still need to be improved. Corpse decomposition is a continuous and complex process that is affected by many factors, such as the corpse itself and the external environment. For example, the process is influenced by individual factors (such as height, body mass, and age) and external factors (such as temperature, soil acidity, and insect activity). Moreover, in forensics, the time and ambient temperature at which the body is discovered are uncontrollable. Therefore, animals, including mice and rats, have been used as models of human cadaver decomposition. In our study, the corpses of SD rats were kept in a controlled environment for 59 days. As expected, corpse stiffness disappeared after 16 hours, and the odour became stronger. Beginning on day 3, trunk and abdomen gas accumulated and led to rupture, and putrid liquid flowed out. Throughout the process of corpse decomposition, the weight of the body decreased daily, which is consistent with the observation of Li et al. (2021).

There are a variety of bacteria in the mouth, which is considered to be the second most complex microbiome of the human body (Adserias‐Garriga et al., 2017). After death, internal and external bacteria act on the body, resulting in internal and external decomposition. During the process of decay, microbiota exhibit a distinct and temporal shift. Although several studies have shown that the oral microbiota can be a useful tool for estimating the PMI (Dong et al., 2019; Eriksson et al., 2017), researchers still know little about the changes in oral bacteria over long time periods. The present study investigated the relationship between oral bacteria and time of death under specific conditions (temperature 25.18 ± 0.75°C; humidity 55.55 ± 14.74% [mean ± SD]) during a 59‐day process of decomposition.

The results showed a particular shift in microbial community richness at different taxon levels, with a downwards trend in the first 24 days and an upwards trend at the remaining time points. A previous study reported that under different or highly variable thermal environmental conditions, the bacterial richness of the pig mouth and skin decreased within 5 days, consistent with the results of our study. Notably, at the early stage, microbial community richness was irrelevant to species and environmental conditions (Pechal et al., 2014). We also found that the oral microbial community of rats in each group changed greatly. At the phylum level, *Proteobacteria*, *Firmicutes*, *Bacteroidetes* and *Actinobacteria* were dominant at all time points.

On day 1, the dominant phyla in the oral cavity community were *Proteobacteria*, *Firmicutes* and *Actinobacteria*, which reached their peak abundance within 1 h after death. *Enterococcus* was dominant at the genus level and was slightly more abundant than *Acinetobacter*, indicating that categories of bacteria in the community did not change immediately after death, which is similar to previous reports (Dong et al., 2019; Huttenhower et al., 2012). In addition, the genus of obligate aerobic bacteria *Acinetobacter* was dominant in group AMH0, while the facultative anaerobic bacteria *Enterococcus* and *Staphylococcus* increased. *Acinetobacter* maintained a high relative abundance, indicating that insufficient oxygenated blood is caused by a decrease in redox potential, which is consistent with the results of previous studies (Vass et al., 2002). A large number of studies have shown that proteolytic enzymes and lipolytic enzymes produced by *Acinetobacter* have an impact on the flavour and smell of dairy products. However, some species of *Acinetobacter* might be opportunistic pathogens (Gurung et al., 2013; Mahnaz et al., 2014; Pangallo et al., 2014). A study by Pechal et al. (2014) suggested that *Acinetobacter* belongs to the Moraxellaceae family and showed a significant level within 24 h of decomposition.

After the above stages, obligate anaerobic bacteria in *Bacteroides* increased at day 5 of the early stage, indicating that a large amount of oxygen entered from the outside due to the rupture of the corpse. *Bacteroides fragilis* belongs to *Bacteroides* and is an opportunistic pathogen whose expression level may reach 40% in the intestines of healthy individuals (Allen et al., 2019). It had a high relative abundance in group AMD5, indicating that it may originate from the intestinal flora. At the genus level, *Bacteroides* and *Acinetobacter* exhibited high relative abundances before day 5 and thus might serve as initial indicators. Consistently, *Cerasibacillus* and *Anaerosalibacter* showed high levels at the late stage after death (Liu et al., 2020; Pechal et al., 2014). Interestingly, *Anaerosalibacter* plays a key role in the fermentation of douchi (a traditional Chinese food) under harsh conditions (Yang et al., 2019). During the decomposition of cadavers, the dynamic changes in bacterial community structure may be due to very low oxygen and humidity, while a high salt concentration is beneficial to bacterial resistance and survival.

In the process of sampling, we found that the corpses emitted an odour after 8 h, indicating that the fat had begun to decompose, which is consistent with the findings of previous studies (Janaway et al., 2009). The facultative anaerobe *Proteus* is capable of fermenting carbohydrates into mixed acid and showed a significant reduction after 5 days, suggesting that carbohydrates tended to decrease over time. Most members of Bacillaceae participate in the carbon, nitrogen, and sulphur cycles in natural environments (Mandic‐Mulec et al., 2015). After 24 days, Bacillaceae displayed an upwards trend, especially *Cerasibacillus*, indicating that members of Bacillaceae were the main taxa able to degrade residues at later stages. *Ignatzschineria*, belonging to Gammaproteobacteria, were the dominant bacteria on day 5, day 10 and day 15. They represent potential indicators for specific time points. In line with our study, Guo J. et al found that a large number of *Ignatzschineria* appeared in the later stage of corruption (Guo et al., 2016). In addition, *Vagococcus* was identified as the crucial indicator genus by several methods (Johnson et al., 2016). *Enterococcus faecalis* is an intestinal symbiotic bacterium that is rarely found in the mouth (Bolocan et al., 2019). In our study, *E. faecalis* was present in the mouth, indicating that a large number of *E. faecalis* were transferred from the intestine to the mouth within 1 day after rat death. This is supported by the findings of previous studies (Melvin et al., 1984), suggesting that the internal bacterial community is more affected by biochemical processes. *Proteus mirabilis* is a urease‐producing bacterium that hydrolyses urea and increases pH (Stickler et al., 2006). This explains why urea can be smelled after the fifth day of corruption.

To establish the PMI estimation model, the RFR algorithm was applied, and three genera, *Atopostipes*, *Facklamia* and *Cerasibacillus*, were screened out. The results suggested that *Atopostipes* and *Facklamia* had the potential to estimate the PMI throughout the decomposition process. Interestingly, as a facultative anaerobe, *Atopostipes* is found in a traditional Chinese food, Aspergillus‐type douchi (Yang et al., 2019). As an obligate aerobe, *Facklamia* is the commensal organism in the oral cavity and is increased in patients with poor immune status, even those with HIV or cancer (Gahl et al., 2020; Mashima et al., 2017; Mukherjee et al., 2018). *Cerasibacillus*, which was present at high abundance at a late stage, was previously found after rupture (Liu et al., 2020). Recent research has suggested that *Cerasibacillus* participates in the biodegradation of organic materials (Rahmati et al., 2017; Wang et al., 2020). Taken together, these results indicate that *Atopostipes*, *Facklamia* and *Cerasibacillus* may be the best potential candidates of oral bacteria for PMI estimation, and further investigation of the three bacteria is warranted, especially in cases of long‐term decomposition.

The PICRUSt outcomes in this study illustrated that energy metabolism was more abundant on day 3 than on day 1, on day 24 and at other time points, indicating that the dominant bacteria on day 3 were mainly involved in this process. A previous study found that the primary bacteria on the third day after death were *Bacteroidetes* and *Firmicutes*, which can ferment amino acids and peptides into propionate and butyrate to produce odour (Scott et al., 2006). The carbohydrate metabolism pathway surged significantly on day 3 and day 24 post‐mortem compared with day 10 and day 15. It has been reported that this pathway is related to increases in the concentrations of hydrogen, carbon dioxide, H_2_S and methane. All of the abovementioned products could facilitate body swelling and odour release 3 and 24 days after death (Louis et al., 2014). Compared with that at other time points, the abundance of *Clostridium sporogenes* on the 59th day after death was relatively high (2.84%). This bacterium can convert tryptophan (an essential amino acid) into indole propionic acid (IPA), which is a component of unpleasant odours (Wikoff et al., 2009).

This study showed that the microbial community structure in the oral cavity changed greatly during the process of cadaver corruption. By detecting the structure of the microbial flora, we found that the richness of phyla, genera and species of the microbial community decreased first and then increased, and the turning point occurred on the 24th day. Through a random forest algorithm, an informative model with three genera of bacteria (*Atopostipes*, *Facklamia* and *Cerasibacillus*) was constructed. This model was helpful in revealing the change trend of oral microorganisms in the process of corpse corruption, especially over the long term.

Although this study provides results of PMI estimation for a long period after death, there are still some limitations. Firstly, it is not tested under more complex conditions, such as different temperatures (high temperature, low temperature) or different humidity; Secondly, there is no human sample verification, which needs to be tested in future research.

Taken together, our findings provide a foundation for analysing the oral bacterial community at specific time points after death. However, some validation work, especially in the human body, needs to be pursued in future research.

## CONFLICT OF INTEREST

The authors declare that there are no conflict of interests.

## Supporting information


Figure S1

Figure S2

Figure S3

Figure S4

Table S1

## Data Availability

The datasets used and/or analyzed during the current study available from the corresponding author on reasonable request.
