# Supplementary figures and images for: Estimation of the post‐mortem interval by modelling the changes in oral bacterial diversity during decomposition
Source: J Appl Microbiol. 2022 Sep 9;133(6):3451–64. doi: 10.1111/jam.15771 (PMC9825971; doi:10.1111/jam.15771)

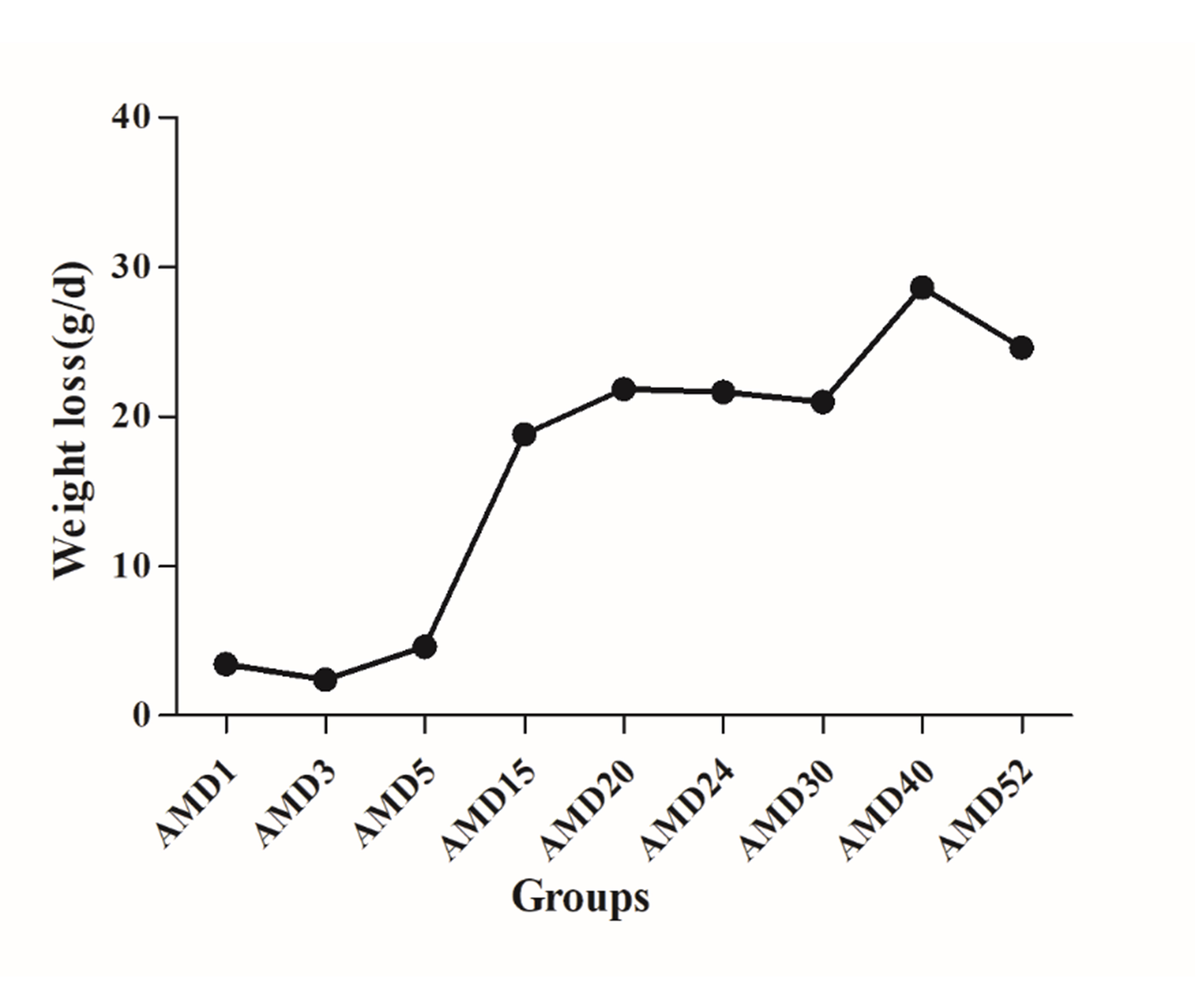

Supplement: Supplementary file 1 — Figure S1 [file JAM-133-3451-s005.jpg]

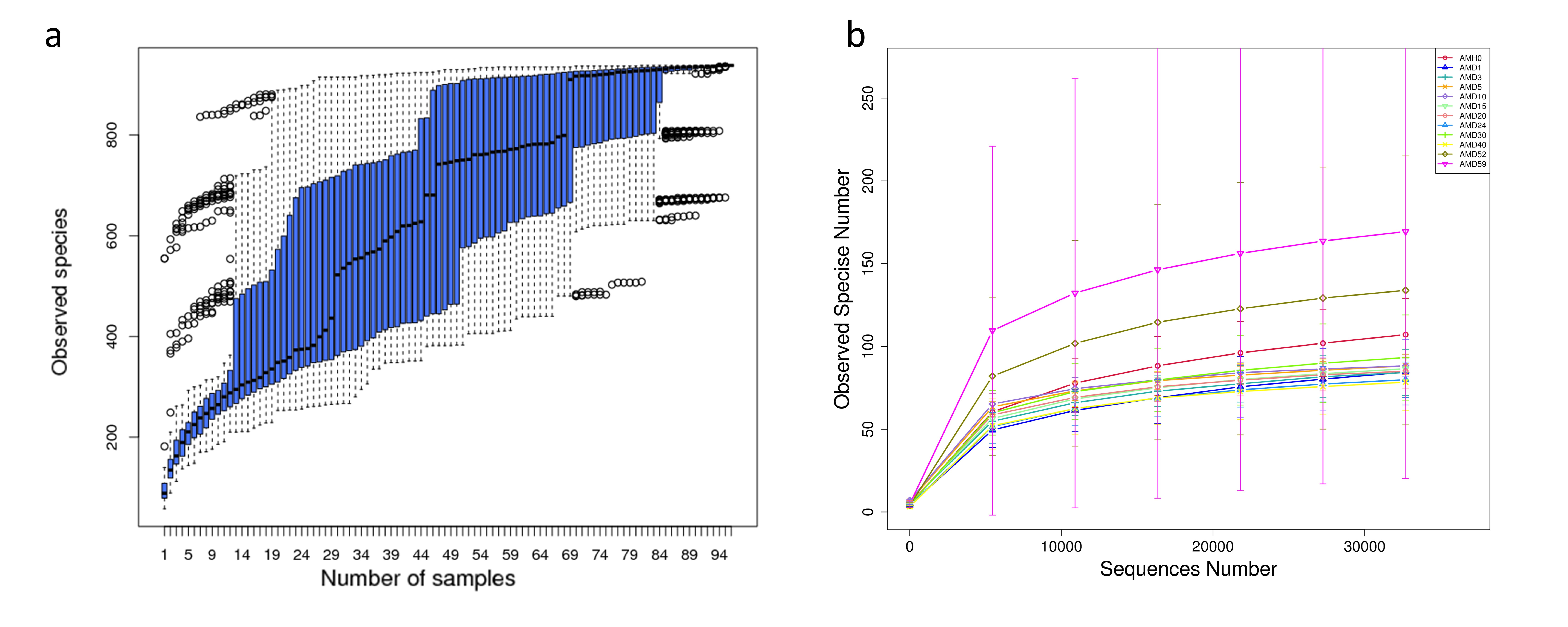

Supplement: Supplementary file 2 — Figure S2 [file JAM-133-3451-s001.jpg]

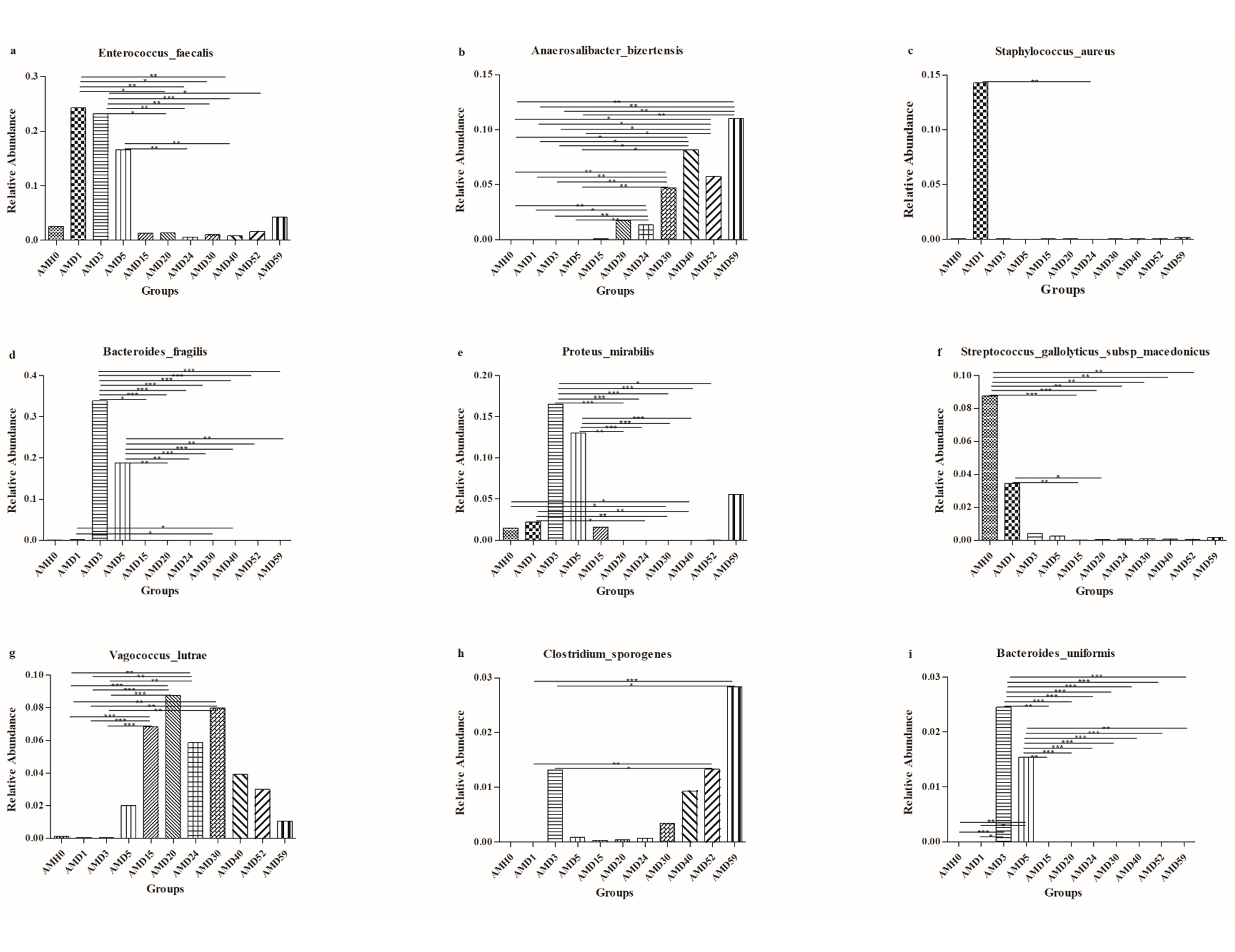

Supplement: Supplementary file 3 — Figure S3 [file JAM-133-3451-s004.jpg]

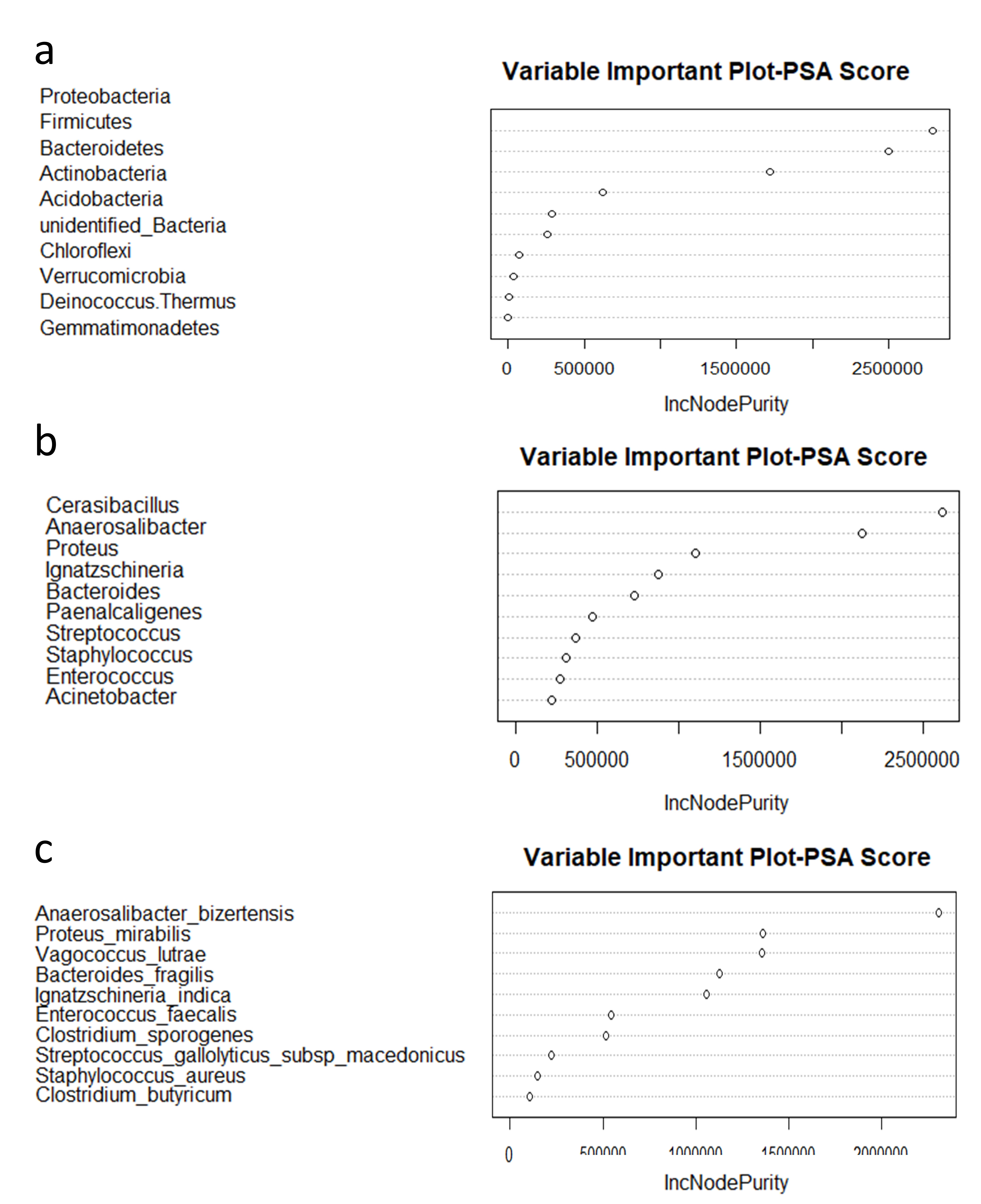

Supplement: Supplementary file 4 — Figure S4 [file JAM-133-3451-s002.jpg]
