# Supplementary material for: Estimation of the post‐mortem interval by modelling the changes in oral bacterial diversity during decomposition
Source: J Appl Microbiol. 2022 Sep 9;133(6):3451–64. doi: 10.1111/jam.15771 (PMC9825971; doi:10.1111/jam.15771)
Supplement: Supplementary file 5 — Table S1 [file JAM-133-3451-s003.docx]

**Table S1** Significant difference of phylum, genus, species microbiota among time points

| Bacteria | | Relative abundance | | | | | | | | | | | | | | | | | | | | | | | |
| --- | --- | --- | --- | --- | --- | --- | --- | --- | --- | --- | --- | --- | --- | --- | --- | --- | --- | --- | --- | --- | --- | --- | --- | --- | --- |
|  |  | AMH0 | | AMD1 | | AMD3 | | AMD5 | | AMD10 | | AMD15 | | AMD20 | | AMD24 | | AMD30 | | AMD40 | | AMD52 | | AMD59 | |
| **Phylum** | |  | |  | |  | |  | |  | |  | |  | |  | |  | |  | |  | |  | |
| *Firmicutes* | | 0.2318 | | 0.4338 | | 0.2950 | | 0.2459 | | 0.2589 | | 0.1011 | | 0.6509 | | 0.9905^*▽◆^ | | 0.9514^*◆^ | | 0.9887^*▼▽◆^ | | 0.9647^*◆^ | | 0.8532^*◆^ | |
| *Proteobacteria* | | 0.7013 | | 0.5623 | | 0.2998 | | 0.5234 | | 0.7402 | | 0.8982 | | 0.3486 | | 0.0078^*▽◆^ | | 0.0443^*▽◆^ | | 0.0098^*▲▽◆^ | | 0.0231^*▽◆^ | | 0.1237^*▽◆^ | |
| *Bacteroidetes* | | 0.0013 | | 0.0021 | | 0.4036 | | 0.2300 | | 0.0006**^△^** | | 0.0004**^△^** | | 0.0000**^*▲△▼^** | | 0.0000**^▲△▼^** | | 0.0001**^▲△▼^** | | 0.0000**^△▼^** | | 0.0006 | | 0.0110**^△^** | |
| *Actinobacteria* | | 0.0651 | | 0.0016 | | 0.0015 | | 0.0007 | | 0.0001**^*^** | | 0.0004**^*^** | | 0.0005**^*^** | | 0.0017**^*^** | | 0.0041 | | 0.0015**^*^** | | 0.0075 | | 0.0079 | |
| **Genus** | |  | |  | |  | |  | |  | |  | |  | |  | |  | |  | |  | |  | |
| *Ignatzschineria* | | 0.0023 | | 0.0023 | | 0.0149 | | 0.2505**^*^** | | 0.6542**^*▲△^** | | 0.8774**^▲^** | | 0.3485 | | 0.0077^◆^ | | 0.0440^◆^ | | 0.0097^◆^ | | 0.0159^▽◆^ | | 0.0068^▽◆^ | |
| *Cerasibacillus* | | 0.0000 | | 0.0001 | | 0.0000 | | 0.0000 | | 0.0000 | | 0.0002 | | 0.0041 | | 0.0217**^*▲△▼^** | | 0.2663**^*▲△▼▽^** | | 0.3485**^*▲△▼▽^** | | 0.3625**^*▲△▼▽^** | | 0.3787**^*▲△▼▽^** | |
| *Acinetobacter* | | 0.2247 | | 0.1968 | | 0.0030 | | 0.0014 | | 0.0000**^*▲△▼^** | | 0.0007 | | 0.0000**^*▲△▼^** | | 0.0000**^*▲△▼^** | | 0.0000**^*▲△▼^** | | 0.0000**^*▲△▼^** | | 0.0000**^*^** | | 0.0000**^*^** | |
| *Enterococcus* | | 0.0274 | | 0.2480 | | 0.2643^*^ | | 0.2049 | | 0.1292 | | 0.0202 | | 0.0171**^▲△▼^** | | 0.0100**^▲△▼^** | | 0.0151**^▲△▼^** | | 0.0094**^▲△▼▽^** | | 0.0195 | | 0.0492 | |
| *Anaerosalibacter* | | 0.0000 | | 0.0000 | | 0.0000 | | 0.0000 | | 0.0000 | | 0.0008 | | 0.0176**^*▲△▼^** | | 0.0135**^*▲△▼▽^** | | 0.0468**^*▲△▼▽^** | | 0.0816**^*▲△▼^** | | 0.0576**^*▲△▼▽^** | | 0.1105**^*▲△▼▽^** | |
| *Staphylococcus* | | 0.0050 | | 0.1462 | | 0.0011 | | 0.0005**^▲^** | | 0.0006 | | 0.0005**^▲^** | | 0.0005**^▲^** | | 0.0002**^▲△^** | | 0.0008 | | 0.0007 | | 0.0010 | | 0.0019 | |
| *Bacteroides* | | 0.0007 | | 0.0017 | | 0.3992 | | 0.2279 | | 0.0006 | | 0.0004 | | 0.0000**^△▼^** | | 0.0000**^△▼^** | | 0.0000**^*△▼^** | | 0.0000**^*△▼^** | | 0.0000**^△▼^** | | 0.0000**^△▼^** | |
| *Streptococcus* | | 0.1613 | | 0.0366 | | 0.0067 | | 0.0044 | | 0.0016**^*▲△^** | | 0.0003**^*▲△^** | | 0.0005**^*▲^** | | 0.0008**^*▲^** | | 0.0010**^*▲^** | | 0.0007**^*▲^** | | 0.0007**^*^** | | 0.0024 | |
| *Proteus* | | 0.0198 | | 0.0295 | | 0.2440 | | 0.1822 | | 0.0204 | | 0.0180 | | 0.0001**^△▼^** | | 0.0000**^*▲△▼^** | | 0.0000**^*▲△▼▽^** | | 0.0000**^*▲△▼^** | | 0.0003**^△^** | | 0.0611 | |
| *Paenalcaligenes* | | 0.0002 | | 0.0004 | | 0.0273 | | 0.0745 | | 0.0000**^▲△^** | | 0.0001 | | 0.0000**^▲△^** | | 0.0000**^▲△^** | | 0.0000**^▲△^** | | 0.0000**^▲△^** | | 0.0000**^▲△^** | | 0.0000**^▲△^** | |
| **Species** | |  | |  | |  | |  | |  | |  | |  | |  | |  | |  | |  | |  | |
| *Enterococcus faecalis* | | 0.0235 | | 0.2262 | | 0.2218^*^ | | 0.1558 | | 0.0712 | | 0.0123 | | 0.0124**^▲△▼^** | | 0.0055**^▲△▼^** | | 0.0095**^▲△▼^** | | 0.0073**^▲△▼^** | | 0.0148**^▲△^** | | 0.0392 | |
|  | |  | |  | |  | |  | |  | |  | |  | |  | |  | |  | |  | |  | |
| *Anaerosalibacter bizertensis* | 0.0000 | | 0.0000 | | 0.0000 | | 0.0000 | | 0.0000 | | 0.0008 | | 0.0176**^*▲△▼^** | | 0.0135**^*▲△▼▽^** | | 0.0468**^*▲△▼▽^** | | 0.0815**^*▲△▼^** | | 0.0576**^*▲△▼▽^** | | 0.1104**^*▲△▼▽^** | |  |
| *Staphylococcus aureus* | 0.0005 | | 0.1428 | | 0.0004 | | 0.0002 | | 0.0004 | | 0.0003 | | 0.0003 | | 0.0001**^▲^** | | 0.0005 | | 0.0004 | | 0.0006 | | 0.0015 | |  |
| *Bacteroides fragilis* | 0.0007 | | 0.0016 | | 0.3405 | | 0.1878 | | 0.0005 | | 0.0003**^△^** | | 0.0000**^△▼^** | | 0.0000**^△▼^** | | 0.0000**^▲△▼^** | | 0.0000**^▲△▼^** | | 0.0000**^△▼^** | | 0.0000**^△▼^** | |  |
| *Proteus mirabilis* | 0.0098 | | 0.0152 | | 0.1046 | | 0.1060 | | 0.0146 | | 0.0124 | | 0.0001**^△▼^** | | 0.0000**^*▲△▼^** | | 0.0000**^*▲△▼▽^** | | 0.0000**^▲△▼^** | | 0.0002**^▼^** | | 0.0509 | |  |
| *Streptococcus gallolyticus subsp. macedonicus* | 0.0876 | | 0.0345 | | 0.0041 | | 0.0025 | | 0.0015**^*▲^** | | 0.0002**^*▲^** | | 0.0004**^*▲^** | | 0.0006**^*^** | | 0.0009**^*^** | | 0.0006**^*^** | | 0.0005**^*^** | | 0.0018 | |  |
| *Ignatzschineria indica* | 0.0004 | | 0.0003 | | 0.0027 | | 0.0412 | | 0.1854**^*▲△^** | | 0.1607**^*▲△^** | | 0.0565 | | 0.0014 | | 0.0069^▽^ | | 0.0014^▽◆^ | | 0.0021^▽◆^ | | 0.0010^▽◆^ | |  |
| *Vagococcus lutrae* | 0.0011 | | 0.0004 | | 0.0005 | | 0.0204 | | 0.0598**^*▲△^** | | 0.0690**^*▲△^** | | 0.0881**^*▲△^** | | 0.0588**^*▲△^** | | 0.0798**^*▲△^** | | 0.0395 | | 0.0300 | | 0.0106 | |  |
| *Clostridium sporogenes* | 0.0000 | | 0.0000 | | 0.0132 | | 0.0009**^*▲△^** | | 0.0129 | | 0.0003 | | 0.0004 | | 0.0007 | | 0.0034 | | 0.0093 | | 0.0133**^*▲^** | | 0.0283**^*▲^** | |  |

*（AMH0）、▲（AMD1）、△（AMD3）、▼（AMD5）、▽（AMD10）、◆（AMD15）、◇（AMD20）compared with other groups showed significant difference.
